# Freiburg Neuropathology Case Conference: A 51-year-old Patient Presenting with Epistaxis and Occasional Headaches 16 Years after Diagnosis of a Grade 1 Chondrosarcoma of the Left Petrous Apex

**DOI:** 10.1007/s00062-023-01294-y

**Published:** 2023-05-12

**Authors:** E. Wogram, F. Schlunk, M. J. Shah, M. Prinz, H. Urbach, D. Erny, C. A. Taschner

**Affiliations:** 1 Department of Neuropathology, Medical Centre, University of Freiburg, Freiburg, Germany; 2 Department of Neuroradiology, Medical Centre, University of Freiburg, Breisacherstraße 64, 79106 Freiburg, Germany; 3 Department of Neurosurgery, Medical Centre, University of Freiburg, Freiburg, Germany; 4 Faculty of Medicine, Medical Centre, University of Freiburg, Freiburg, Germany

**Keywords:** Chondrosarcoma, Chordoma, Skull base metastasis, Skull base meningioma, Radiologic-pathologic correlation

## Case Report

**Fig. 1:**
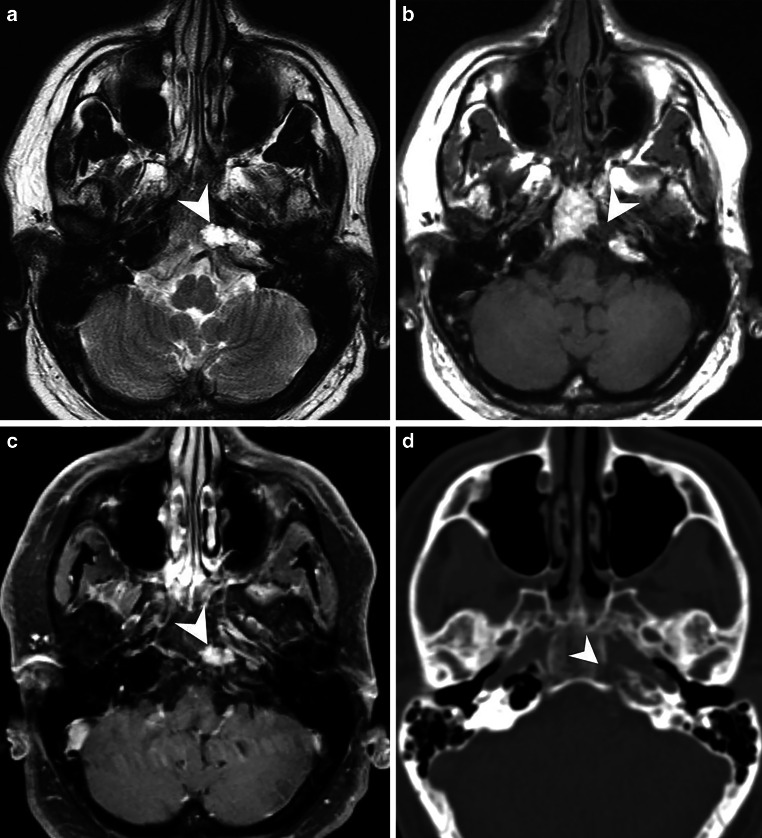
Magnetic resonance (MR) and computed tomography (CT) scans performed at the time of initial presentation 16 years ago. Axial T2-weighted images (**a**) showed a clearly demarcated lesion (*arrowhead*) at the apex of the left petrous bone, which had a homogeneous hyperintense signal. On axial T1-weighted images, the lesion appeared hypointense (**b**, *arrowhead*). On T1-weighted images after administration of gadolinium (Gd), the lesion showed marked and homogeneous contrast enhancement (**c**, *arrowhead*). On axial CT images in bone window settings, the lesion showed marked osteolysis (*arrowhead*). The histologic diagnosis at that time was grade 1 chondrosarcoma

Sixteen years ago, a then 35-year-old male patient presented with a right-sided flickering scotoma, diffuse vertigo, a tendency to fall to the left, and nausea. The patient had a history of recurrent headaches with retrobulbar accentuation. Magnetic resonance imaging (MRI) and computed tomography (CT) showed an osteolytic process in the left petrous apex (Fig. 1). Osteoplastic suboccipital trepanation was performed on the left side, and a biopsy was obtained. No tumor was found in the histologic specimen and 3 months after surgery, the pre-existing headache recurred. Therefore, it was decided to perform another transnasal transphenoidal biopsy. Histologically, a low-grade chondroid tumor was diagnosed, corresponding to a differentiated grade 1 chondrosarcoma and 6 months later, proton-ion radiotherapy with 60 Gy followed.

**Fig. 2:**
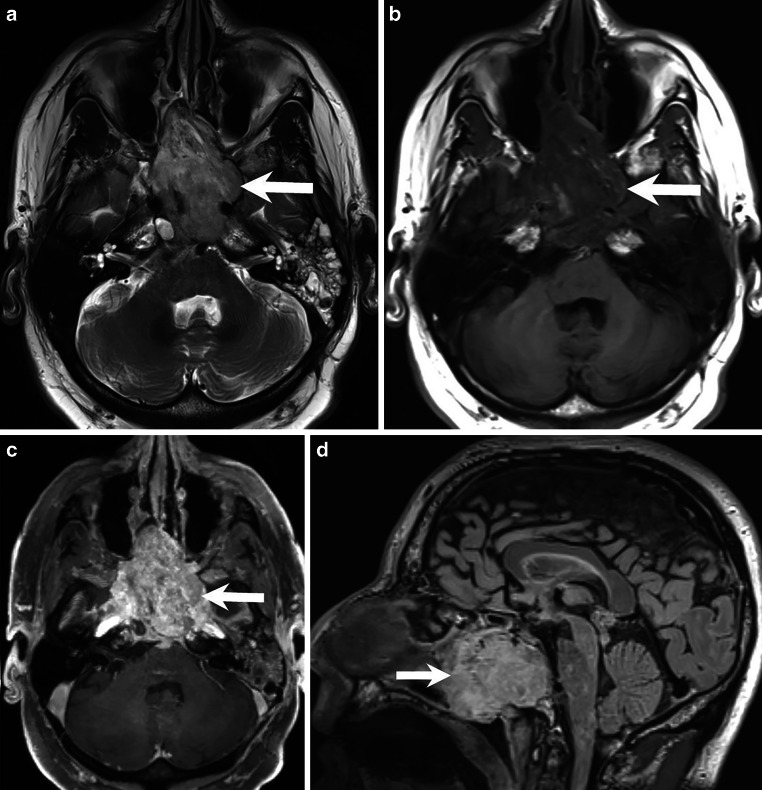
Magnetic resonance and CT images after tumor progression and increasing clinical symptoms over the past 2 years. On axial T2-weighted images, the hyperintense mass lesion infiltrated the sphenoid sinus, encircled the left internal carotid artery, and had direct contact with the basilar artery (**a**, *arrow*). On axial T1-weighted images, the lesion appeared hypointense (**b**, *arrow*). On axial T1-weighted images acquired after administration of gadolinium, the lesion appeared with marked and fairly homogeneous contrast enhancement (**c**, *arrow*). Sagittal fluid attenuated inversion recovery (FLAIR) images showed the size of the extended mass lesion (**d**, *arrow*)

**Fig. 3:**
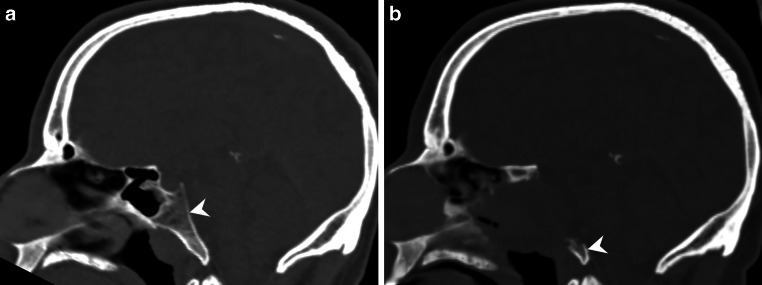
Sagittal reconstructions of CT images in bone window settings at initial presentation (**a**) and at the time of the last biopsy (**b**) show the osteodestructive features of the lesion with almost completely destroyed clivus (**a** + **b**, *arrowhead*)

Both clinically and with regular MRI checks, the situation was stable for 10 years. In the twelfth year after irradiation, MRI showed changes suggestive of local recurrence (Figs. 2 and 3). Because of clinical symptoms (headache and nosebleeds) and tumor progression on subsequent MR controls, a total of three inconclusive extended biopsies were performed on different occasions via a transnasal approach. A final, this time conclusive, transnasal biopsy was performed in December 2022, and the patient was discharged under stable conditions. Subsequently, a new proton ion radiotherapy was initiated.

## Imaging

Magnetic resonance (MR) and computed tomography (CT) scans performed at the time of initial presentation 16 years ago showed a lesion in the apex of the left petrous bone, adjacent to the clivus (Fig. 1). On T2-weighted images, the lesion appeared clearly demarcated and showed a homogeneous hyperintense signal (Fig. 1a, arrowhead). On T1-weighted images, the lesion appeared hypointense (Fig. 1b, arrowhead). After administration of gadolinium (Gd), the lesion showed marked and homogeneous contrast enhancement (Fig. 1c, arrowhead). On CT images with bone window settings, the lesion showed marked osteolysis (Fig. 1d, arrowhead). The histologic diagnosis at that time was a differentiated grade 1 chondrosarcoma.

Magnetic resonance and CT images obtained after tumor progression and increasing symptoms over the past 2 years showed an extensive osteodestructive mass lesion of the skull base (Figs. 2 and 3). On T2-weighted images, the hyperintense mass lesion infiltrated the sphenoid sinus, encircled the left internal carotid artery, and had direct contact with the basilar artery (Fig. 2a, arrow). On T1-weighted images, the lesion appeared hypointense (Fig. 2b, arrow) with marked contrast enhancement after administration of Gd (Fig. 2c, arrow). Sagittal fluid attenuated inversion recovery (FLAIR) images showed the size of the extensive mass lesion. Sagittal reconstructions of CT images in the bone window at initial presentation (Fig. 3a) and at the time of the last biopsy (Fig. 3b) showed the osteodestructive features of the lesion with the clivus (Fig. 3a, arrowhead) almost completely destroyed (Fig. 3b, arrowhead).

## Differential Diagnosis

### Chondrosarcoma

Chondrosarcoma of the skull base is a rare malignant tumor, arising from chondroid cells and accounting for approximately 0.15% of all intracranial neoplasms [1]. The tumor, which affects mainly middle-aged adults, is typically located off-midline in the petroclival region and spheno-ethmoidal sinus [2]. Location is determined by the embryology because the bones of the skull base develop by endochondral ossification and remnants of these cells may undergo malignant transformation. Although commonly slow growing, more aggressive WHO grade II and III lesions occur [3]. Clinical presentation varies with the exact location and involvement of anatomical structures but often includes headache and cranial nerve palsy [3]. Radiologic diagnosis is challenging due to the resemblance of the tumor to other entities, such as chordoma or skull base metastasis. Skull base chondrosarcomas typically appear isointense or hypointense on T1‑w images and hyperintense on T2‑w images. Most but not all lesions show heterogeneous contrast enhancement [4]. A recent study reported DWI and dynamic contrast enhanced perfusion MR imaging as useful sequences to distinguish chondrosarcoma from other skull base tumors [5]. The CT imaging is useful to visualize bony involvement as well as calcification, which is present in approximately 50% of patients and often has a characteristic ring-like and arc-like configuration [6].

In the present case, because of the location of the tumor as well as the imaging characteristics with high T2‑w imaging signal and contrast enhancement, the initial imaging findings made chondrosarcoma the most probable diagnosis.

### Chordoma

Chordomas and the abovedescribed chondrosarcomas of the skull base share many similarities. While chordomas are in comparison a more frequent tumor entity, they still account for less than 1% of all intracranial tumors only [2, 7]. The tumor derives from remnants of the notochord, and is therefore typically centered in the midline, which is a possible differentiating factor from chondrosarcomas. The median age at diagnosis is around 60 years; however, it can affect any age group [8]. Chordoma has an aggressive growth pattern and a high recurrence rate despite multimodal treatment. Symptoms relating to mass effect on surrounding structures include headache, diplopia or cranial nerve impairment [2]. On MR imaging, chordomas demonstrate high T2‑w signal due to high fluid content [9]. Small foci of hemorrhage or calcium are possible findings within the tumor. While contrast enhancement is variable and does not serve to distinguish chordomas from chondrosarcomas and metastasis of the skull base, lower apparent diffusion coefficient (ADC) levels have been reported for chordomas [5]. The CT scans reveal a well-circumscribed, expansile soft tissue mass that arises from the clivus with associated extensive lytic bone destruction [10].

In our patient, the tumor was not located exactly in the midline; however, because of the matching MR characteristics we considered chordoma a valid differential diagnosis.

### Skull Base Metastases

Skull base metastases account for approximately 1–5% of all brain metastases [11]. The incidence varies depending on the primary malignancy, with lung and breast cancers being the most common primary sites. Other neoplasms that can manifest in the skull base include melanoma, renal cell carcinoma, gastrointestinal cancer, and prostate cancer [12]. Skull base metastases affect males and females equally and most often occur in older age groups, with a peak incidence at 65 years and older [13]. The location of skull base metastasis is highly variable. The most common site of osseous metastasis is the petrous apex and the clivus, due to high amounts of bone marrow [12, 14]. Skull base metastasis can be in multiple compartments and affect bones as well as dura and leptomeninges at the same time [15]. Clinical presentation varies greatly depending on location and extent of the mass and can include headaches, cranial neuropathy, or can be asymptomatic [16]. Imaging of an enhancing destructive mass of the skull base in a patient with a known primary malignancy makes metastases the most likely diagnosis. The CT scans reveal lytic, sclerotic or mixed type lesions of cortical and trabecular bone [14]. Skull base metastasis can show diffusion restriction and are regularly enhanced on postcontrast MRI scans. On T1‑w imaging lesions in the bone marrow are hypointense and replace high signal fat [17, 18].

The absence of a known malignancy and the singularity of the lesion made skull base metastasis more unlikely in our patient. Initial imaging could not rule out this differential diagnosis completely, however.

### Skull Base Meningioma

The most common tumors of the skull base are meningiomas, accounting for approximately 20–25% of all intracranial tumors [19, 20]. Patients are most commonly middle-aged to old individuals and women are affected more often [21]. The tumor originates from arachnoid cap cells and is in 90% of cases benign [22]; however, in a minority of cases meningiomas are higher grade tumors (WHO grade II and grade III) [23]. They are typically located in the midline, and can frequently be found in the olfactory groove, tuberculum sellae, sphenoid wing, petroclival and cavernous sinuses as well as the foramen magnum [22, 24]. Similar to the conditions described above, clinical presentation varies depending on location and extent of the tumor, and can include headache, cranial nerve deficits such as vision disturbance, and seizures [22, 25]. Imaging features include a well-circumscribed, homogeneously and vividly enhancing mass on postcontrast imaging with possible bony involvement. Meningiomas are usually hypointense to isointense to grey matter on T1‑w and T2‑w images [24]. The CSF vascular cleft sign between tumor and parenchyma proves the extra-axial location. A dural tail sign is a typical feature of skull base meningioma but not present in all cases. CT scans can demonstrate hyperostosis or sclerotic adjacent bone at the site of the tumor [26].

The high T2 signal in the present case, as well as the absence of a dural attachment on imaging made this diagnosis unlikely in our patient.

## Histology and Immunohistochemistry

The first specimen obtained from the patient in February 2007 from the petrous apex measured 1 mm^3^ and exhibited bone fragments, connective tissue, and blood cells. No indication of inflammation or neoplasm was evident.

**Fig. 4:**
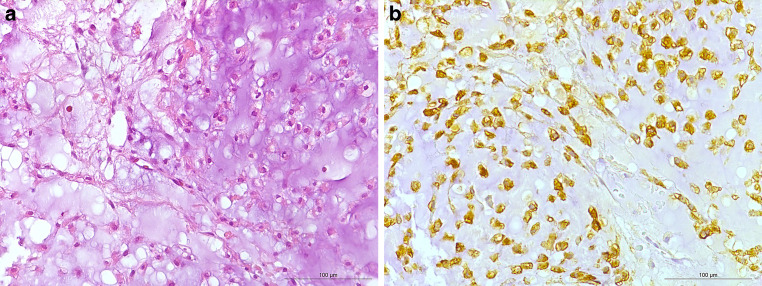
Analysis of the initial histopathological specimen from the patient revealed numerous features, such as minimally increased cellularity and nodular growth distinctive to a grade 1 chondrosarcoma. Hematoxylin-eosin (H&E) stained section (**a**) showed cartilaginous matrix with chondrocytes embedded in lacunae with minimally increased cellularity. A few tumor cells reacted positively in the immunohistochemistry for S100 (**b**) (scale bars: 100 µm)

The patient’s second histopathologic specimen in August 2007 showed features characteristic of grade I chondrosarcoma, including minimally increased cellularity and nodular growth (Fig. 4a). No mitotic figures were evident. Based on immunohistochemistry, the malignant cells did not express brachyury, but S100 (Fig. 4b). This ruled out the differential diagnosis chordoma. The diagnosis of a grade 1 chondrosarcoma was confirmed at the bone tumor reference center, University Hospital of Basel.

**Fig. 5:**
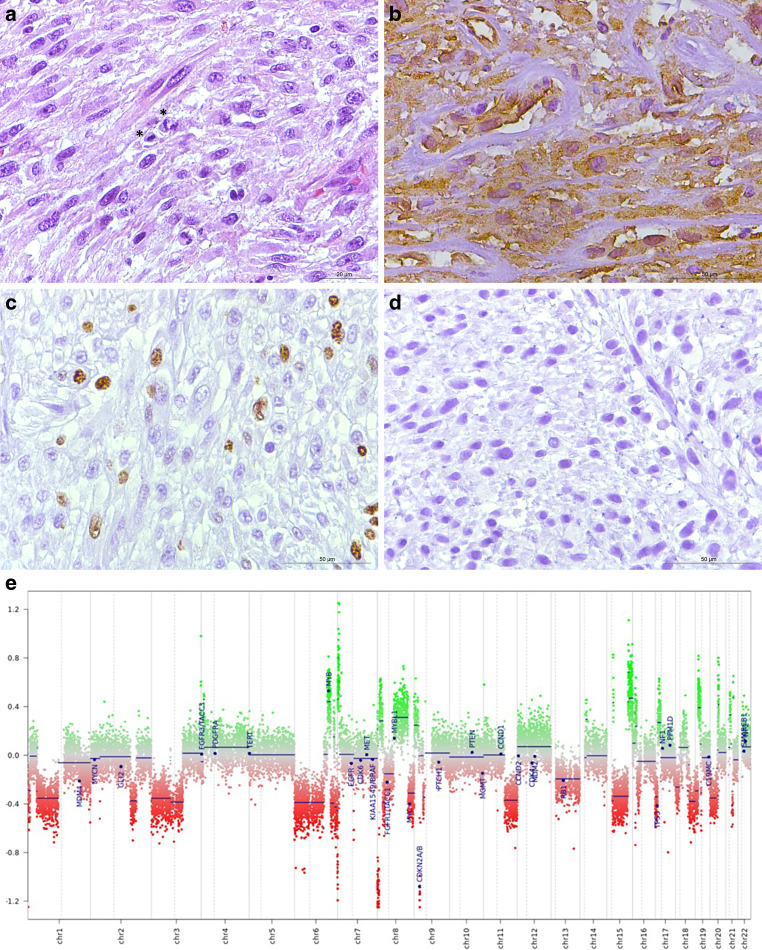
Sixteen years after the initial diagnosis an expansive tumor mass had developed within the same location. The tumor was surgically removed and examined. Immunohistochemistry for CD68 (**a**) depicted a vast number of myeloid cells. Tumor cells reacted positively in the immunohistochemistry for vimentin (**b**). Immunohistochemistry for Mib1 (**c**) showed a vast amount of proliferating tumor cells. Immunohistochemistry for brachyury (**d**) showed no positive staining. Scale bars: 50 µm. 850k DNA methylation assay of the tumor tissue (**e**) matched the pattern of the methylation class for undifferentiated sarcoma

In December 2022, a tumor from the sphenoid sinus was surgically removed and examined. The histopathological analysis revealed numerous spindle-shaped cells with mitotic figures (Fig. 5a). Immunohistochemistry revealed that the neoplastic cells were negative for pan-cytokeratin, EMA, Desmin, and S100, and positive for vimentin (Fig. 5b). A few tumor cells showed a positive reaction in the immunohistochemistry for SMA (not shown). Ki-67 was detected by immunohistochemistry (Mib1) in approximately 25% of the tumor cells (Fig. 5c), the tumor cells did not express brachyury (Fig. 5d). The histopathological examination was complemented by an 850k DNA methylation assay of the tumor tissue [27]. The methylation pattern was compared to a reference dataset, the brain tumor classifier, version 12.2. The methylation pattern of the tumor tissue matched the pattern of the methylation class undifferentiated sarcoma (Fig. 5e). Notably, the assay revealed a CDKN2A/B deletion, an observation reported before in the context of soft tissue sarcoma [28]. Additionally, sequencing of IDH‑1 and IDH‑2 revealed wild-type sequences. For further evaluation, the samples were evaluation at the bone tumor reference center, at the University Hospital of Basel, Switzerland. There, the diagnosis of undifferentiated high-grade sarcoma was confirmed.

## Diagnosis

### Undifferentiated High-grade Sarcoma, IDH-Wildtype

Radiation-induced sarcomas, particularly undifferentiated pleomorphic sarcomas, may occur as a long-term complication of radiotherapy [29]. In cancers of the head and neck, the reported risk of developing radiation-induced sarcomas in long-term survivors is 0.3% [30]. Because the present patient underwent radiotherapy, the pathology observed in the last specimen may well be radiotherapy-induced.

On the other hand, numerous chondrosarcoma cases have been described that have dedifferentiated to a tumor resembling a high-grade sarcoma, including undifferentiated pleomorphic sarcomas [31]. Dedifferentiation into anaplastic lesions is expected in 11% of chondrosarcomas [32]. To test this differential diagnosis, we examined features common to chondrosarcomas, i.e., an IDH1 or IDH2 mutation described in approximately 38.7% and 12.1% of chondrosarcoma specimens, respectively. DNA sequencing did not reveal an IDH1 or IDH2 mutation [33]. Therefore, it was not possible to attribute the current pathology to the original chondrosarcoma on the basis of the IDH1 or IDH2 mutation. Consequently, the differential diagnosis of dedifferentiated chondrosarcoma cannot be excluded.
